# Is the association of overweight and obesity with colorectal cancer underestimated? An umbrella review of systematic reviews and meta-analyses

**DOI:** 10.1007/s10654-022-00954-6

**Published:** 2023-01-21

**Authors:** Marko Mandic, Hengjing Li, Fatemeh Safizadeh, Tobias Niedermaier, Michael Hoffmeister, Hermann Brenner

**Affiliations:** 1 Division of Clinical Epidemiology and Aging Research, German Cancer Research Center (DKFZ), Im Neuenheimer Feld 581, 69120 Heidelberg, Germany; 2 Institute for Medical Information Processing, Biometry and Epidemiology - IBE, LMU Munich, Munich, Germany; 3 Pettenkofer School of Public Health, Munich, Germany; 4 Medical Faculty Heidelberg, Heidelberg University, Heidelberg, Germany; 5 Division of Preventive Oncology, German Cancer Research Center (DKFZ), National Center for Tumor Diseases (NCT), Heidelberg, Germany; 6 German Cancer Consortium (DKTK), German Cancer Research Center (DKFZ), Heidelberg, Germany

**Keywords:** Colorectal cancer, Body mass index, Prediagnostic weight loss, Obesity

## Abstract

Although high body-mass index (BMI) is associated with increased risk of developing colorectal cancer (CRC), many CRC patients lose weight before diagnosis. BMI is often reported close to diagnosis, which may have led to underestimation or even reversal of direction of the BMI-CRC association. We aimed to assess if and to what extent potential bias from prediagnostic weight loss has been considered in available epidemiological evidence. We searched PubMed and Web of Science until May 2022 for systematic reviews and meta-analyses investigating the BMI-CRC association. Information on design aspects and results was extracted, including if and how the reviews handled prediagnostic weight loss as a potential source of bias. Additionally, we analyzed how individual cohort studies included in the latest systematic review handled the issue. Overall, 18 reviews were identified. None of them thoroughly considered or discussed prediagnostic weight loss as a potential source of bias. The majority (15/21) of cohorts included in the latest review did not exclude any initial years of follow-up from their main analysis. Although the majority of studies reported having conducted sensitivity analyses in which initial years of follow-up were excluded, results were reported very heterogeneously and mostly for additional exclusions of 1–2 years only. Where explicitly reported, effect estimates mostly increased with increasing length of exclusion. The impact of overweight and obesity on CRC risk may be larger than suggested by the existing epidemiological evidence.

**Supplementary Information:**

The online version contains supplementary material available at 10.1007/s10654-022-00954-6.

## Introduction

Overweight and obesity, commonly defined by a body-mass index (BMI) ≥ 25 to 30 kg/m^2^ and ≥ 30 kg/m^2^[1], respectively, are established risk factors for a variety of cancers including colorectal cancer (CRC) [2]. Recent reviews have indicated that overweight and obese individuals have about 18% and 32% greater risk of CRC compared to those with normal weight [3–5]. However, a large 2017 meta-analysis reported even stronger associations with colorectal adenomas, the precursors of most CRCs, with a risk increase by more than 40% for both overweight and obesity [6]. Furthermore, there is evidence that CRC patients may experience substantial weight loss in the preclinical phase prior to diagnosis [7], suggesting that the strength of the association of overweight and obesity with CRC risk may have been underestimated in epidemiological studies due to prediagnostic weight loss. In case–control studies, BMI is often reported for the time close to or shortly before diagnosis among cases. In such studies, prediagnostic weight loss may have led to underestimation or even reversal of direction of the BMI-CRC association [8, 9]. For example, Low et al. [8] showed that being overweight or obese at diagnosis is associated with a 31% reduction of early-onset CRC risk. In contrast to that, their post-hoc analyses suggested that the cases were more likely to have had a significant reduction in weight in the 5-year period before diagnosis. In cohort studies with BMI ascertained at baseline, underestimation of the BMI-CRC association may also be of concern as cancers diagnosed during the early years of follow-up may have been present in preclinical state already at enrollment and may have led to weight loss before enrollment. Mean sojourn time in preclinical state has been estimated to be around 3–6 years for CRC [10–12]. Tumor-associated weight loss due to preclinical CRC or its precursors may again have led to an underestimation of the BMI-CRC association unless the initial years of follow-up were excluded from the analysis.

This study aims to evaluate if and to what extent the association of overweight and obesity with CRC may have been underestimated in epidemiological studies by the aforementioned sources of bias. Therefore, we conducted an umbrella review and searched for reviews that investigated the BMI-CRC association and to examine if and how these reviews and the included studies in those reviews handled potential bias due to prediagnostic weight loss.

## Methods

Our study protocol was registered with PROSPERO at inception (registration number: CRD42021256462). Changes made during the review were recorded in PROSPERO. We followed standardized methodology guidelines summarized in the Preferred Reporting Items for Systematic Reviews and Meta-Analyses (PRISMA)(Supplementary File 1) [13].

### Search strategy and selection criteria

We systematically searched PubMed and Web of Science from inception through 9^th^ May 2022 for systematic reviews and meta-analyses that investigated the association between overweight and obesity with risk of colorectal cancer using a predefined search algorithm. Search terms included ‘colorectal cancer’, ‘body-mass index’, ‘risk factor’ (or related terms), combined with ‘systematic review’*,* and ‘meta-analysis’ (or related terms, see Supplementary File 2 for the details of the algorithms used for both databases). The reference lists of identified studies were also searched for additional relevant studies. We excluded letters, editorials, comments, news, and articles published in languages other than English.

Studies were eligible for inclusion if they were a systematic review or a meta-analysis study, and if the primary exposure of interest was obesity or overweight (defined by BMI, (weight in kilograms) / (height in meters)^2^) and if the primary outcome was colorectal cancer or its anatomic subsites, rectal or colon cancer. Studies that exclusively used different measures of adiposity (e.g. waist-to-hip ratio, weight, weight gain, etc.) were not included.

### Data extraction and evaluation of study quality

Data extraction was performed independently by two authors (MM and HL). Initial disagreements were resolved by consensus after further review and discussion. From each study, the following information was extracted: First author’s name, article title, publication year, number of included studies (grouped by study type), exposure definition, summary effect size estimates (most adjusted) and their 95% CIs, model type, and measures of heterogeneity (I^2^ statistic or Q-test’s *p*-value). Where possible, a combined effect estimate for colon and rectal cancer was extracted, otherwise, both effect size estimates were reported. The same was done for sex-specific/combined effect size estimates. However, to ensure comparability of summary estimates from different reviews and easier interpretation of forest plots, we performed a generic inverse-variance random-effects meta-analysis for studies that only reported sex- or site-specific estimates.

The methodological quality of each systematic review was independently assessed by two investigators (MM and HL) using AMSTAR-2 (A Measurement Tool to Assess Systematic Reviews) [14], and initial disagreements were again resolved by consensus after further review and discussion. AMSTAR-2 is a validated and reliable measurement tool consisting of 16 items and includes ratings for the quality of the search, reporting, transparency, and statistical analysis (Supplementary File 3). According to the suggestions in AMSTAR-2 guidelines, items 2, 4, 7, 9, 11, 13, and 15 were defined as critical domains, and items 1, 3, 5, 6, 8, 10, 12, 14, and 16 were defined as non-critical domains. The final rating criteria were defined as follows: high quality when one or no non-critical weaknesses were found, moderate when two or more non-critical weaknesses were found, low quality when one critical weakness with or without non-critical weaknesses was found, and critically low quality when two or more critical weaknesses with or without non-critical weaknesses were found.

Potential bias arising from prediagnostic weight loss was ascertained by two dichotomous (yes/no) items: ‘timing of BMI ascertainment’ was used as a criterion for case–control studies and was rated as ‘considered’ in the review/meta-analysis if the exact timing of BMI in each of the primary studies was reported, and if timing was considered in the analysis and estimation of the summary effects (through stratification/subgroup analysis/exclusion of studies with BMI ascertainment too close to diagnosis). ‘Consideration of sojourn time’ was used as a criterion for cohort studies and was rated as ‘considered’ in the reviews/meta-analyses if they presented summary analyses for cohorts in which at least the first 4 years of follow-up were excluded.

To further investigate how individual primary studies handled potential bias due to prediagnostic weight loss, we extracted the information from the individual studies included in the most recent systematic review, which only included cohort studies. The information we recorded was if and how many of the first years of follow-up were excluded in the main analyses or potential sensitivity analyses. From studies that expanded the exclusion of the first years of follow-up in sensitivity analysis, we recorded and compared hazard ratios and their 95% CIs from main and sensitivity analyses. All analyses and data visualization were conducted using R version 4.1.1.

## Results

### Characteristics of the included studies

**Fig. 1:**
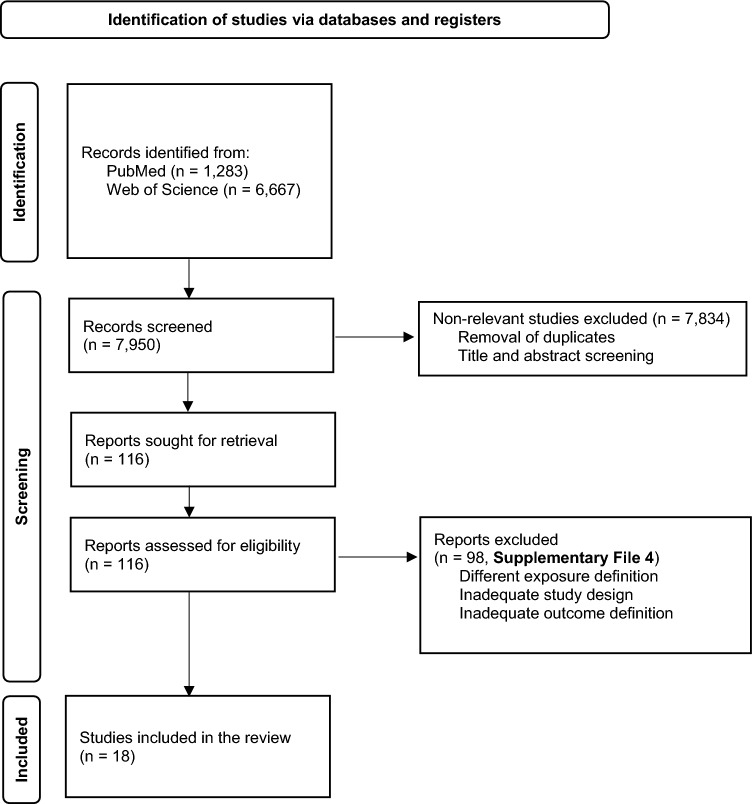
Preferred Reporting Items for Systematic Reviews and Meta-Analyses (PRISMA) flow diagram

Overall, a total of 7,950 articles were retrieved from the systematic search in two databases. In the end, 18 publications met all inclusion criteria (Fig. 1) [2–5, 15–28].

Table 1 provides an overview of the characteristics of the included reviews. Of these 18 reviews, 13 (72%) included only cohort studies (prospective or retrospective), while the remaining 5 included both cohort and case–control studies. Summary estimates of relative risk were most commonly reported for the risk of obesity compared to normal weight according to the WHO definition (obesity: BMI ≥ 30 kg/m^2^; overweight: 25 kg/m^2^ ≤ BMI < 30 kg/m^2^; normal: 18.5 kg/m^2^ ≤ BMI < 25 kg/m^2^; underweight: BMI < 18.5 kg/m^2^) and for the risk associated with a 5 kg/m^2^ BMI increase. Three reviews reported results for ‘High versus Low BMI’ (based on included primary studies), one review reported relative risk per 8 kg/m^2^ increase in BMI, and another review reported results for an increase in BMI by one standard deviation.

We assessed the methodological quality of 17 reviews (1 publication was not a systematic review) using the AMSTAR-2 tool (Supplementary File 3). Almost all (16/17, 94%) of the assessed studies had two or more critical flaws [mostly in item 7, “Did the review authors provide a list of excluded studies and justify the exclusions?” (15/17, 88%), and item 13, “Did the review authors account for risk of bias in individual studies when interpreting/ discussing the results of the review?” (14/17, 82%)]. Therefore, most of the reviews had a critically low quality score, except Fang et al. 2018 [28], which had a moderate quality score. Timing of the BMI ascertainment and sojourn time consideration were analyzed separately. None of the included reviews fulfilled our predefined criteria. Of the two criteria for assessing the risk of bias due to timing of the BMI ascertainment, only Ning and colleagues (2010) [19] fulfilled the first criterion by reporting the exact time-point at which the BMI was recorded in the primary studies. Sojourn time was not analyzed nor discussed in any of the reviews.

### Overview of summary results from systematic reviews and meta-analyses

**Fig. 2:**
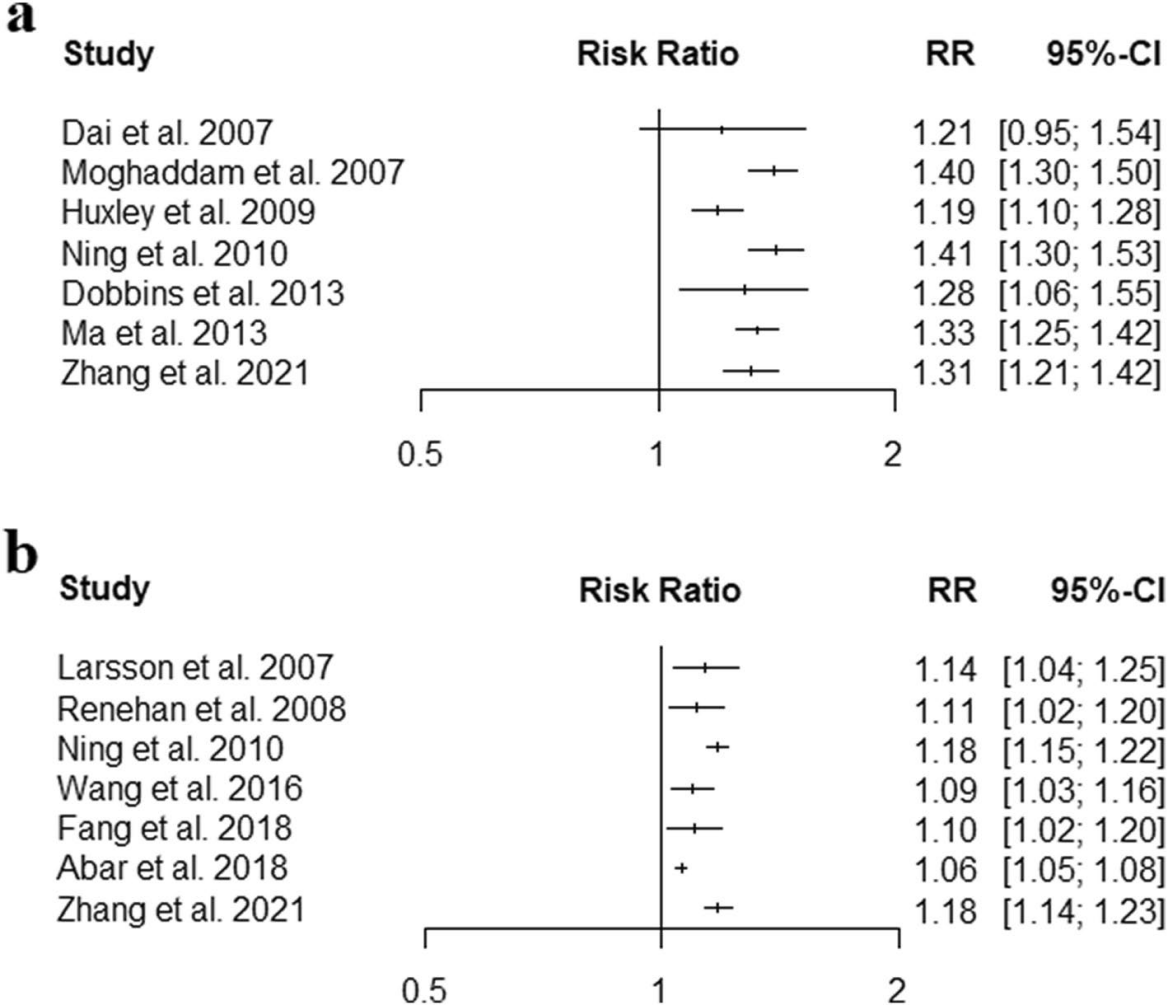
Forest plot of the summary estimates from the reviews with meta-analyses in the umbrella review of the association between CRC risk and **a)** obesity (BMI ≥ 30 kg/m^2^) in comparison with normal weight (BMI ≥ 18.5 kg/m^2^ and BMI < 25.0 kg/m^2^); **b)** 5 kg/m^2^ increase in BMI BMI = body-mass index, CC = colon cancer, CI = confidence interval, CRC = colorectal cancer, F = female, M = male, RC = rectal cancer, RR = relative risk

Since the included reviews reported risk estimates for different definitions of exposure, we focused on the two most common definitions: WHO definition of obesity (BMI ≥ 30 kg/m^2^) in comparison to normal weight (BMI ≥ 18.5 kg/m^2^ and < 25.0 kg/m^2^), and 5 kg/m^2^ increments in BMI. Furthermore, due to expected differences between colon cancer and rectal cancer risk, site-specific results are presented separately for both outcomes. Summary results of the different meta-analyses are presented by forest plots. Due to the overlap of studies included in the meta-analyses, we refrained from summarizing the summary estimates of association by meta-analysis. Figure 2A shows the summary risk estimates for the association between obesity and CRC. Summary estimates of relative risk for both sexes and both sites combined ranged from 1.19 to 1.41, and were reported as 1.33 (95% CI 1.25 to 1.42) and 1.31 (95% CI 1.12 to 1.42) in the two most recent meta-analyses. Figure 2B shows the summary risk estimates for the association between 5 kg/m^2^ increments in BMI and CRC. Relative risks for both exposure definitions were consistently higher for men than for women, and for colon cancer than for rectal cancer in subgroup- and site-specific meta-analyses (Supplementary Figures S1 and S2).

### More detailed assessment of the sojourn times in the most recent review of cohort studies

**Fig. 3:**
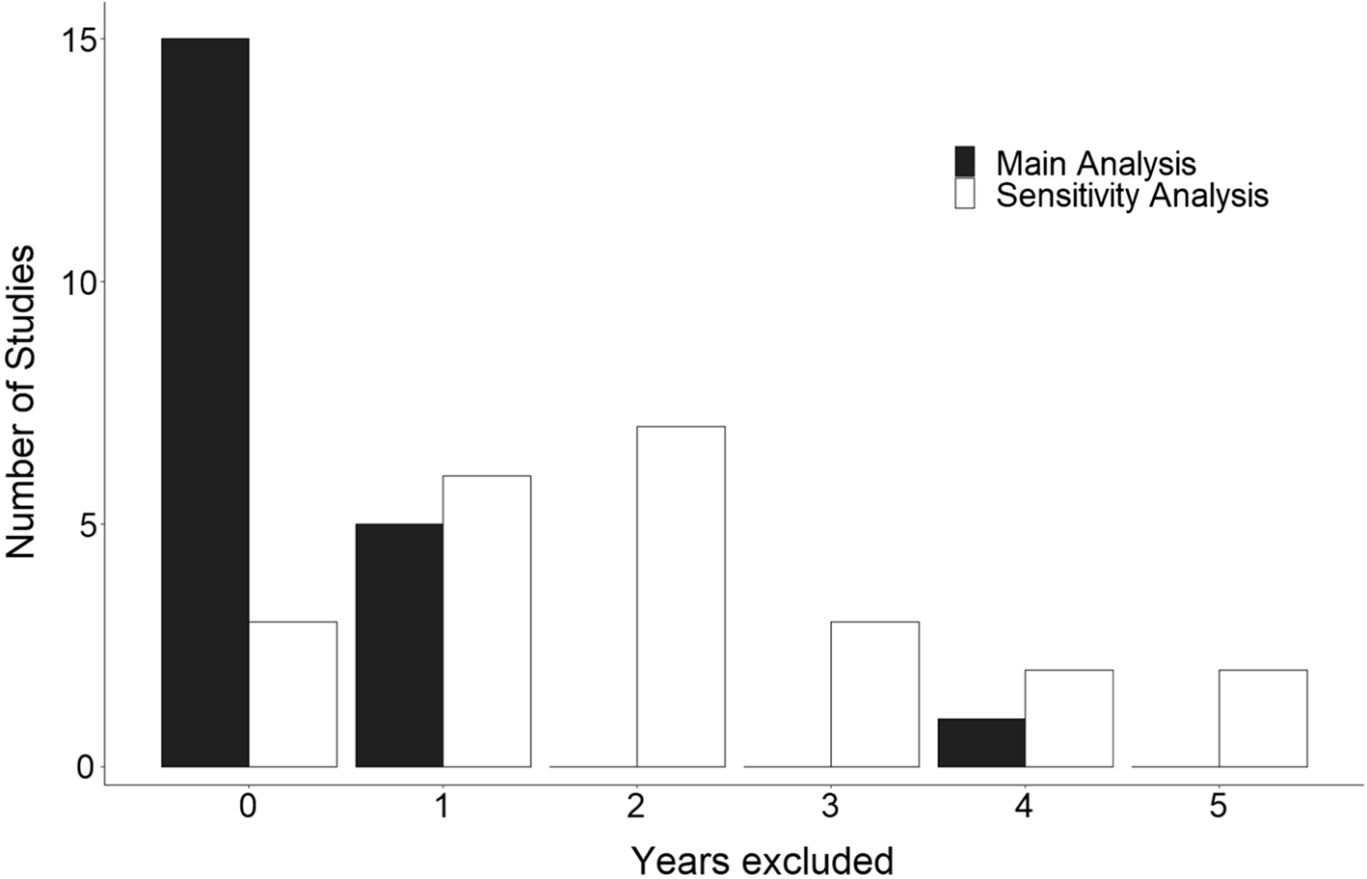
First years of follow-up excluded in the main and sensitivity analyses in the individual studies included in Zhang et al. (2021)^3^

An overview of how individual cohort studies (those included in the most recent review by Zhang et al. 2021) [3] handled the issue of sojourn time is presented in Supplementary Table 1 and Fig. 3 [29–54]. In the main analysis, more than two-thirds of the studies (15/21, 71%) did not exclude any of the first years of follow-up, 5 excluded the first year, and one excluded the first 4 years of follow-up. In the sensitivity analysis, two-thirds (14/21) did not exclude more than the first 2 years of follow-up. Hazard ratios from studies that have excluded more years in their sensitivity analysis than in their main analysis and have explicitly reported their estimates, are shown in Supplementary Table 2. The majority of the studies (12/15) extended their exclusion of the initial follow-up years by just 1 or 2 years. There was very high heterogeneity in the details of reporting. Some studies only stated that the results did not change significantly in the sensitivity analysis, and some reported only the estimates that reached statistical significance. On the other hand, few studies reported all of the results of their sensitivity analysis. In these studies, estimates from the sensitivity analyses were somewhat larger than the corresponding estimates in the main analyses in most cases.

## Discussion

It is well established that overweight and obesity are important risk factors for many cancers, including CRC [2]. To the best of our knowledge, this is the first umbrella review to systematically investigate how previous systematic reviews and meta-analyses, as well as the primary studies included in the most recent systematic review, approached prediagnostic weight loss as a potential source of bias. Our analysis suggests that more rigorous attention to this potential source of bias may be needed to fully disclose the impact of overweight and obesity on CRC risk.

Sojourn time for CRC in the preclinical state has consistently been estimated to be about 3–6 years [10–12]. This prediagnostic period often goes along with weight loss [7, 55, 56]. Since most of the case–control studies ascertained BMI close to the time of diagnosis, such studies, and systematic reviews and meta-analyses that included them, are likely to have underestimated the relationship between overweight and obesity and CRC. In initial analyses, Low et al. (2020) showed that increased BMI was associated with a significant reduction in the odds for early-onset CRC (ORs: 0.69 (95% CI 0.55–0.87) and 0.69 (0.55–0.86) for overweight and obesity, respectively). While this study focused on early-onset CRC, there is no reason to assume this would be different for CRC at older ages. Post-hoc analyses showed that cases had a higher chance of experiencing considerable weight loss than controls (ORs: 2.23 (95% CI 1.76–2.83) for a weight loss ≥ 5 kg and 2.50 (1.65–3.78) for weight loss ≥ 10 kg). This means that the initial estimates are, as the authors explained, most likely strong underestimates of the true relationship between excess weight and CRC.

Although most of the reviews and meta-analyses identified in our umbrella review included only prospective cohort studies, the lack of or very limited exclusion of the initial years of follow-up, even in sensitivity analyses, in the original studies suggests that both the individual cohort studies and the meta-analyses again have underestimated the obesity-CRC association. In none of the systematic reviews was this major concern thoroughly addressed in the discussion.

A closer look at the primary studies included in the most recent systematic review and meta-analysis (Zhang et al. 2021) [3] reveals that, in the main analysis, 15 out of 21 studies (71%) did not implement any sort of exclusion of the first years of follow-up, and even where sensitivity analyses were reported, they rarely excluded more than the first one or 2 years of follow-up. Furthermore, even if conducted, such sensitivity analyses varied strongly in the details of reporting. For example, Bhaskaran et al. [43] and Song et al. [36] thoroughly reported the results of their sensitivity analyses. Most other studies only partially and selectively reported sensitivity analysis results, e.g. when a nonsignificant estimate between BMI and CRC risk reached statistical significance, while others simply stated that the results remained similar, without reporting the actual estimates. In most of the few studies that explicitly reported sensitivity analyses, the effect estimates increased, albeit mostly to a limited extent, possibly due to the short periods of exclusion. Concerns about bias due to prediagnostic weight loss are even more salient in meta-analyses which were consistently based on the results of the main analyses (with typically no or minimal exclusion of initial years of follow-up) rather than the results of the sensitivity analyses.

Most of the reviews/meta-analyses reported a positive association of BMI with CRC. For obesity, relative risk summary estimates for both sexes and both sites ranged from 19 to 41% increased CRC risk. However, a 2017 systematic review and meta-analysis of 168,201 subjects evaluating the association of investigator-measured body-mass index and colorectal adenomas, the precursors of most CRCs, revealed even strong estimates for both overweight and obesity: 44% (95% CI 30–61%) and 42% (95% CI 24–63%) increased risk compared to normal weight [6]. For a 5 kg/m^2^ increase in BMI, summary estimates in the reviews were in the range of 6% to 18% increased CRC risk (combined for both sexes and both sites). Summary estimates were significantly higher for CC than for RC. The World Cancer Research Fund (WCRF) and the American Institute for Cancer Research (AICR) Continuous Update Project 2018 report includes a particularly low summary estimate of 5% (95% CI 3–7%) increase in CRC risk per 5-unit BMI increment [57]. This report is widely regarded as a trusted and authoritative scientific resource due to its most rigorous methodology. Nevertheless, like all of the meta-analyses published in peer-reviewed journals identified in our umbrella review, this estimate is expected to be affected by lack of or very limited precautions against bias due to disease-related weight loss of the included cohort studies and may therefore likewise underestimate the BMI-CRC association.

The estimates of relative risk of CRC associated with overweight and obesity from epidemiological studies and their meta-analyses are commonly used to calculate the fraction of CRC cases in the population that is attributable to overweight and obesity. These so-called population-attributable fractions additionally depend on the prevalence of overweight and obesity in the population. Given the high and increasing prevalence of overweight and obesity, even modest increases in risk of CRC translate into relatively high shares of CRC cases statistically attributable to overweight and obesity. For example, in Germany, two-thirds of adult men (67%) and half of the women (53%) are overweight or obese (BMI ≥ 25 kg/m^2^), and about a quarter are obese (23% of men, 24% of women) [58]. Prevalences are even higher in the US, where, in 2018, 74% of all adults were either overweight or obese, and 42% were obese [59]. The estimated proportions of CRC cases due to overweight and obesity based on previous estimates of CRC risks from epidemiological studies were already as high as 13.2% for Germany [60]. The corresponding estimate for the US was 5.2%. However, it was based on the previously mentioned very low estimate by the WCRF-AICR [61]. It is expected that these estimates would be substantially higher when taking underestimation of risks due to prediagnostic weight loss into account.

Our study focused on the role of prediagnostic weight loss as a potential source of bias. Although probably being a particularly relevant source of bias, other sources of potential bias also require careful attention. These include, for example, the inaccuracy of weight measures, in particular in studies based on self-reported weight. In cohort studies, where weight is ascertained prior to disease manifestation, less than perfect weight ascertainment would most likely be nondifferential with respect to CRC risk, and therefore further attenuate observed BMI-CRC associations. Other potential sources of biases, such as imperfect control for confounding (despite major attempts of multivariate adjustments commonly made in epidemiological studies) may lead to either over- or underestimation of the BMI-CRC association.

A key strength of this paper is that it includes a comprehensive review of existing systematic reviews and meta-analyses investigating the relationship between BMI and CRC. Nonetheless, our analysis also has several limitations. Firstly, despite a comprehensive, systematic search in multiple databases, we cannot exclude having missed some systematic reviews. However, even if that should be the case, this would not invalidate our findings since we aimed to explore if and how reviews, in general, approached the prediagnostic weight loss problem. Secondly, although we thoroughly reviewed the individual studies included in the most recent systematic review and identified several studies that have, at least to some extent, addressed the issue of prediagnostic weight loss, heterogeneity in reporting of these studies precluded redoing the meta-analysis based on such sensitivity analyses. Thirdly, our study focused exclusively on BMI as an indicator of overweight and obesity. Future studies should also consider alternative indicators, such as waist-to-hip ratio, to investigate if their association with CRC risk might be affected by prediagnostic weight loss in a similar manner.

In conclusion, our umbrella review suggests that the association between overweight and obesity and CRC risk may be stronger than suggested by previous studies and meta-analyses which paid no or only limited attention to prediagnostic weight loss. Future studies should pay more thorough attention and adequately account for lifetime history of weight in order to fully disclose the impact of overweight and obesity on CRC risk. Even though prediagnostic weight loss may be of particular concern for CRC, it may also play an important role for other cancers and some non-cancer diseases whose association with overweight and obesity may also have been underestimated by previous research. Further research should therefore take care of prediagnostic weight loss in an even broader context. Most notably, however, our study underlines the need for enhanced efforts for more effective prevention of overweight and obesity, which have become more prevalent in many countries in the past decades, and which may be stronger risk factors for CRC and possibly also for other cancers and diseases than suggested by existing epidemiological studies.

**Table 1:** Overview of characteristics of the included reviews

First author, year	Number of studies	Study designs	Exposure definition^a^	Outcome	Sojourn time considered	Timing of BMI ascertainment	Summary effect estimate^b^ RR (95% CI)	I^2^ (%) or Q-test (*p*-value)
Dai et al. 2007[15]	15	Cohort Only	Overweight (WHO definition) Obesity (WHO definition)	CRC	No	n/a	M: 1.26 (1.04–1.52) F: 1.09 (0.95–1.26) M: 1.37 (1.21–1.56) F: 1.07 (0.97–1.18)	M: p = 0.42 F: p = 0.29
Larsson et al. 2007[16]	30	Cohort Only	5 kg/m^2^ increase	CC RC	No	n/a	M: 1.30 (1.25–1.35) F: 1.12 (1.07–1.18) M: 1.12 (1.09–1.16) F: 1.03 (0.99–1.08)	M: 30.7 F: 49.2 M: 9.5 F: 13.0
Moghaddam et al. 2007[17]	31	Mixed	BMI ≥ 30 kg/m^2^ vs BMI < 25 kg/m^2^	CRC	No	No	1.40 (1.31–1.51)	p < 0.001
Renehan et al. 2008[2]	29	Cohort Only	5 kg/m^2^ increase	CC RC	No	n/a	M: 1.24 (1.21–1.28) F: 1.09 (1.05–1.14) M: 1.09 (1.06–1.12) F: 1.02 (0.99–1.04)	M: 21.0 F: 39.0 M: 3.0 F: 0.0
Huxley et al. 2009[18]	18	Cohort Only	BMI ≥ 30 kg/m^2^ vs BMI < 25 kg/m^2^	CRC	No	n/a	1.19 (1.11–1.29)	Not reported
Ning et al. 2010[19]	56	Cohort Only	BMI ≥ 30 kg/m^2^ vs BMI < 23 kg/m^2^ 5 kg/m^2^ increase	CRC	No	n/a	1.41 (1.30–1.53) 1.18 (1.14–1.21)	Not reported
Dobbins et al. 2013[20]	16 (M) 13 (F) 11 (M) 9 (F)	Cohort Only	Obesity (WHO definition)	CC RC	No	n/a	M: 1.57 (1.48–1.65) F: 1.19 (1.04–1.36) M: 1.22 (0.91–1.64) F: 1.03 (0.74–1.44)	M: 0.0 F: 39.0 M: 92.0 F: 39.0
Esposito et al. 2013[21]	6	Mixed	‘High BMI’ based on primary studies	CRC	No	No	1.14 (1.04–1.22)	43.4
Johnson et al. 2013[22]	23	Mixed	8 kg/m^2^ increase	CRC	No	No	1.10 (1.08–1.12)	Not reported
Ma et al 2013[23]	41	Cohort Only	‘High BMI’ based on primary studies	CRC	No	n/a	1.33 (1.25–1.42)	68.9
Robsahm et al. 2013[24]	17	Cohort Only	‘High BMI’ based on primary studies	dCC pCC RC	No	n/a	1.59 (1.34–1.89) 1.24 (1.08–1.42) 1.23 (1.02–1.48)	44.0 15.1 21.6
Wang et al. 2016[25]	30	Mixed	5 kg/m^2^ increase	CRC	No	No	M: 1.13 (1.10–1.17) F: 1.06 (1.03–1.09)	M: 72.3 F: 50.4
Freisling et al. 2017[26]	7	Cohort Only	1 s.d. increase	CRC	No	n/a	1.16 (1.04–1.30)	30.5
Abar et al. 2018[27]	38	Cohort only	5 kg/m^2^ increase	CRC	No	n/a	1.06 (1.04–1.07)	83.0
Fang et al. 2018[28]	34	Cohort only	5 kg/m^2^ increase	CRC	No	n/a	M: 1.15 (1.10–1.19) F: 1.06 (1.03–1.10)	M: 78.0 F: 66.7
Garcia et al. 2019[5]	15	Cohort only	‘High BMI’ based on primary studies	CRC	No	n/a	M: 1.39 (1.20–1.62) F: 1.19 (1.06–1.35)	M: 53.2 F: 24.6
Lei et al. 2020[4]	15	Mixed	Overweight (WHO definition) Obesity (WHO definition)	CRC	No	No	1.18 (1.08–1.28) 1.32 (1.11–1.56)	50.2 64.9
Zhang et al. 2021[3]	26	Cohort only	Obesity (WHO definition) 5 kg/m^2^ increase	CRC	No	n/a	1.31 (1.21–1.42) 1.18 (1.14–1.23)	83.9 85.1

BMI = body-mass index, CC = colon cancer, CI = confidence interval, CRC = colorectal cancer, dCC = distal colon cancer, F = female, M = male, n/a = not applicable, pCC = proximal colon cancer, RC = rectal cancer, Ref = reference, RR = relative risk, s.d. = standard deviation, WHO = World Health Organization

^a^‘WHO definition’ refers to the comparison of WHO-defined obesity (BMI ≥ 30.0 kg/m^2^) with normal weight (BMI ≥ 18.5 kg/m^2^ and BMI < 25.0 kg/m^2^). ‘High BMI’ refers to the comparison of obesity with normal weight using different definitions found in primary studies

^b^Summary effect estimates using random-effect models

### Supplementary Information

Below is the link to the electronic supplementary material.

Supplementary file1 (DOCX 125 KB)

Supplementary file2 (DOCX 128 KB)

Supplementary file3 (DOCX 21 KB)

Supplementary file4 (DOCX 14 KB)

Supplementary file5 (PDF 126 KB)

Supplementary file6 (PDF 235 KB)

Supplementary file7 (DOCX 34 KB)

Supplementary file8 (DOCX 29 KB)

## Abbreviations

AICR: American Institute for Cancer Research
BMI: Body-mass index
CC: Colon cancer
CI: Confidence interval
CRC: Colorectal cancer
OR: Odds ratio
RC: Rectal cancer
RR: Relative risk
WCRF: World Cancer Research Fund
WHO: World Health Organization
