# Correction: Is the association of overweight and obesity with colorectal cancer underestimated? An umbrella review of systematic reviews and meta-analyses

**DOI:** 10.1007/s10654-023-01092-3

**Published:** 2024-01-17

**Authors:** Marko Mandic, Hengjing Li, Fatemeh Safizadeh, Tobias Niedermaier, Michael Hoffmeister, Hermann Brenner

**Affiliations:** 1 Division of Clinical Epidemiology and Aging Research, German Cancer Research Center (DKFZ), Im Neuenheimer Feld 581, 69120 Heidelberg, Germany; 2 Institute for Medical Information Processing, Biometry and Epidemiology - IBE, LMU Munich, Munich, Germany; 3 Pettenkofer School of Public Health, Munich, Germany; 4 Medical Faculty Heidelberg, Heidelberg University, Heidelberg, Germany; 5 Division of Preventive Oncology, German Cancer Research Center (DKFZ), National Center for Tumor Diseases (NCT), Heidelberg, Germany; 6 German Cancer Consortium (DKTK), German Cancer Research Center (DKFZ), Heidelberg, Germany

**European Journal of Epidemiology (2023) 38:135-144**

10.1007/s10654-022-00954-6

The wrong Supplementary file 7 was originally published with this article; it has now been replaced with the correct file.

The original article has been corrected.

### Electronic supplementary material

Below is the link to the electronic supplementary material

Supplementary Material 1

