# Supplementary material for: Is the association of overweight and obesity with colorectal cancer underestimated? An umbrella review of systematic reviews and meta-analyses
Source: Eur J Epidemiol. 2023 Jan 21;38(2):135–44. doi: 10.1007/s10654-022-00954-6 (PMC9905196; doi:10.1007/s10654-022-00954-6)
Supplement: Supplementary file 1 — Supplementary file1 (DOCX 125 KB) [file 10654_2022_954_MOESM1_ESM.docx]

**
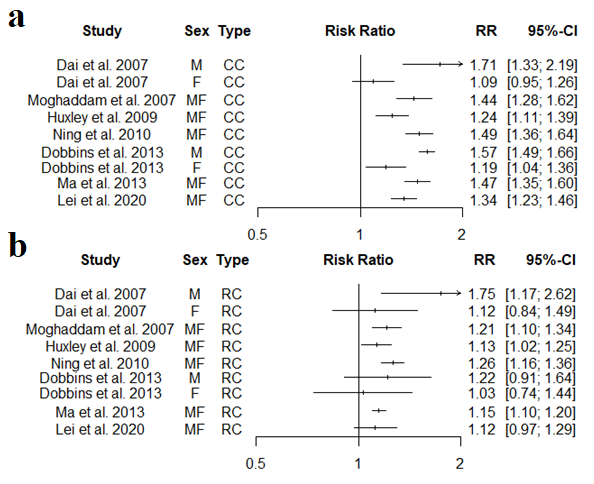
**

**Supplementary Fig S1** Forest plot of the summary estimates from the reviews with meta-analyses in the umbrella review of the association between obesity (BMI ≥30 kg/m^2^ and **a)** CC risk; **b)** RC risk

Abbreviations: BMI = body-mass index, CC = colon cancer, CI = confidence interval, CRC = colorectal cancer, F = female, M = male, RC = rectal cancer, RR = relative risk.
