# Supplementary material for: Is the association of overweight and obesity with colorectal cancer underestimated? An umbrella review of systematic reviews and meta-analyses
Source: Eur J Epidemiol. 2023 Jan 21;38(2):135–44. doi: 10.1007/s10654-022-00954-6 (PMC9905196; doi:10.1007/s10654-022-00954-6)
Supplement: Supplementary file 4 — Supplementary file4 (DOCX 14 KB) [file 10654_2022_954_MOESM4_ESM.docx]

**Search strategies and results**

**Web of Science**

#1

((TS=(((colorect* OR colon OR colonic OR rectal* OR rectum* OR “large bowel”) AND (cancer* OR malignan* OR carcinom* OR tumo* OR CRC OR adenocarcinoma* OR adenom* OR lesion* OR neoplas*)))) AND LA=(English)) AND DT=(Article OR Abstract of Published Item OR Early Access OR Reprint OR Retracted Publication OR Review)

Hits: 292,975

#2

((TS=((bmi OR body-mass OR “body mass” OR “body weight” OR overweight* OR “over weight” OR “body weight” OR “body fat” OR “body fatness” OR “body size” OR obes* OR adipos* OR “waist circumference” OR “hip circumference” OR anthropometr* OR “risk factor” OR “risk factors” OR “weight gain” OR “weight loss” OR “lifestyle factor” OR “lifestyle factors”))) AND LA=(English)) AND DT=(Article OR Abstract of Published Item OR Early Access OR Review OR Retracted Publication OR Reprint)

Hits: 1,539,771

#3

((TS=((“systematic review” OR “systematic reviews” OR “meta-analysis” OR “meta-analyses” OR “meta analysis” OR “meta analyses” OR “systematic overview” OR “systematic overviews”))) AND LA=(English)) AND DT=(Abstract of Published Item OR Article OR Early Access OR Reprint OR Retracted Publication OR Review)

Hits: 1,025,180

#4

((TI=((overview* OR review* OR summary* OR risk*))) AND LA=(English)) AND DT=(Abstract of Published Item OR Article OR Early Access OR Retracted Publication OR Review OR Reprint)

Hits: 2,667,389

#5

TI=(animal* OR “animal experiment” OR “animal model” OR “animal tissue” OR “non human” OR nonhuman OR rat OR rats OR mice OR mouse OR swine OR porcine OR murine OR sheep OR lambs OR pig OR pigs OR piglet* OR rabbit* OR monkey OR bovine)

#6

(#1 AND #2 AND (#3 OR #4) NOT #5)

Hits: 6,667

**PubMed**

#1

(colorect*[Title/Abstract] OR colon[Title/Abstract] OR colonic[Title/Abstract] OR rectal*[Title/Abstract] OR rectum*[Title/Abstract] OR “large bowel”[Title/Abstract]) AND (cancer*[Title/Abstract] OR malignan*[Title/Abstract] OR carcinom*[Title/Abstract] OR tumo*[Title/Abstract] OR CRC[Title/Abstract] OR adenocarcinoma*[Title/Abstract] OR adenom*[Title/Abstract] OR lesion*[Title/Abstract] OR neoplas*[Title/Abstract])

Hits: 306,627

#2

(bmi[Title/Abstract] OR body-mass[Title/Abstract] OR “body mass”[Title/Abstract] OR “body weight”[Title/Abstract] OR  overweight*[Title/Abstract] OR  “over weight”[Title/Abstract] OR “body weight”[Title/Abstract] OR “body fat”[Title/Abstract] OR “body fatness”[Title/Abstract] OR “body size”[Title/Abstract] OR obes*[Title/Abstract] OR adipos*[Title/Abstract] OR “waist circumference”[Title/Abstract] OR “hip circumference”[Title/Abstract] OR anthropometr*[Title/Abstract] OR “risk factor”[Title/Abstract] OR “risk factors”[Title/Abstract] OR “weight gain”[Title/Abstract] OR “weight loss”[Title/Abstract] OR “lifestyle factor”[Title/Abstract] OR “lifestyle factors”[Title/Abstract])

Hits: 1,551,953

#3

(“systematic review”[Title/Abstract] OR “systematic reviews”[Title/Abstract] OR “meta-analysis”[Title/Abstract] OR “meta-analyses”[Title/Abstract] OR “meta analysis”[Title/Abstract] OR “meta analyses”[Title/Abstract] OR “systematic overview”[Title/Abstract] OR “systematic overviews”[Title/Abstract])

Hits: 360,402

#4

overview*[Title] OR review*[Title] OR summary*[Title]

Hits: 681,518

#5

editorial[Publication Type] OR letter[Publication Type] OR Comment*[Publication Type] OR

news[Publication Type]

Hits: 2,252,947

#6

(animal*[Title] OR “animal experiment”[Title] OR “animal model”[Title] OR “animal tissue”[Title] OR “non human”[Title] OR nonhuman[Title] OR rat[Title] OR rats[Title] OR mice[Title] OR mouse[Title] OR swine[Title] OR porcine[Title] OR murine[Title] OR sheep[Title] OR lambs[Title] OR pig[Title] OR pigs[Title] OR piglet*[Title] OR rabbit*[Title] OR monkey[Title] OR bovine[Title])

Hits: 2,043,051

#1 AND #2 AND (#3 OR #4) NOT #5 NOT #6

Hits: 1,283
