# Supplementary material for: Is the association of overweight and obesity with colorectal cancer underestimated? An umbrella review of systematic reviews and meta-analyses
Source: Eur J Epidemiol. 2023 Jan 21;38(2):135–44. doi: 10.1007/s10654-022-00954-6 (PMC9905196; doi:10.1007/s10654-022-00954-6)
Supplement: Supplementary file 5 — Supplementary file5 (PDF 126 KB) [file 10654_2022_954_MOESM5_ESM.pdf]

Supplementary File 3. AMSTAR-2 checklist.

	Item 1	**Item 2**	Item 3	**Item 4**	Item 5	Item 6	**Item 7**	Item 8	**Item 9**	Item 10	**Item 11**	Item 12	**Item 13**	Item 14	**Item 15**	Item 16
Moghaddam et al. 2007[17]	○	●	○	○	●	○	●	○	●	●	○	●	●	○	○	○
Larsson et al. 2007[16]	○	●	○	○	●	●	●	○	●	●	○	●	●	●	○	○
Dai et al. 2007[14]	○	●	○	○	●	●	●	○	●	●	○	●	●	○	○	○
Renehan et al. 2008[2]	○	○	○	○	○	●	○	○	●	●	○	●	●	○	○	○
Huxley et al. 2009[18]	○	●	○	○	●	●	●	○	●	●	○	●	●	○	○	○
Ning et al. 2010[19]	○	●	○	○	●	○	●	○	●	●	○	●	●	○	○	○
Robsahm et al. 2013[24]	○	○	○	○	●	●	●	○	●	●	○	○	○	○	●	○
Ma et al. 2013[23]	○	○	○	○	○	○	●	○	●	●	○	●	●	○	○	○
Johnson et al. 2013[22]	○	●	○	○	●	●	●	●	●	●	○	●	●	○	○	○
Esposito et al. 2013[21]	○	●	○	○	○	○	●	○	●	●	○	●	●	○	○	○
Dobbins et al. 2013[20]	○	●	○	○	○	○	●	○	●	●	○	●	●	●	●	○
Wang et al. 2016[25]	○	●	○	○	○	○	●	○	●	●	○	●	●	○	○	○
Fang et al. 2018[26]	○	○	○	○	○	○	○	○	○	●	○	●	○	○	○	○
Abar et al. 2018[27]	○	○	○	○	●	○	●	○	●	●	○	●	●	○	○	○
Garcia et al. 2019[5]	○	●	○	○	○	○	●	○	○	●	○	●	○	●	○	○
Lei et al. 2020[4]	○	●	○	○	●	○	●	○	○	●	○	●	●	○	○	○
Zhang et al. 2021[3]	○	○	○	○	○	○	●	○	○	●	○	●	●	○	○	○

○ = Yes, ● = No.

Item 1: “Did the research questions and inclusion criteria for the review include the components of PICO?” **Item 2**: "Did the report of the review contain an explicit statement that the review methods were established prior to the conduct of the review and did the report justify any significant deviations from the protocol?" Item 3: “Did the review authors explain their selection of the study designs for inclusion in the review?” **Item 4**: “Did the review authors use a comprehensive literature search strategy?” Item 5: “Did the review authors perform study selection in duplicate?” Item 6 - Did the review authors perform data extraction in duplicate?” **Item 7**: “Did the review authors provide a list of excluded studies and justify the exclusions?” Item 8 - Did the review authors describe the included studies in adequate detail?” **Item 9**: "Did the review authors use a satisfactory technique for assessing the risk of bias (RoB) in individual studies that were included in the review?" Item 10: “Did the review authors report on the sources of funding for the studies included in the review?” **Item 11**: "If meta-analysis was performed did the review authors use appropriate methods for statistical combination of results?" Item 12: "If meta-analysis was performed, did the review authors assess the potential impact of RoB in individual studies on the results of the meta-analysis or other evidence synthesis?" **Item 13**: "Did the review authors account for RoB in individual studies when interpreting/ discussing the results of the review?" Item 14: "Did the review authors provide a satisfactory explanation for, and discussion of, any heterogeneity observed in the results of the review?” **Item 15**: "If they performed quantitative synthesis did the review authors carry out an adequate investigation of publication bias (small study bias) and discuss its likely impact on the results of the review?" Item 16: "Did the review authors report any potential sources of conflict of interest, including any funding they received for conducting the review?".
