# Supplementary material for: Is the association of overweight and obesity with colorectal cancer underestimated? An umbrella review of systematic reviews and meta-analyses
Source: Eur J Epidemiol. 2023 Jan 21;38(2):135–44. doi: 10.1007/s10654-022-00954-6 (PMC9905196; doi:10.1007/s10654-022-00954-6)
Supplement: Supplementary file 6 — Supplementary file6 (PDF 235 KB) [file 10654_2022_954_MOESM6_ESM.pdf]

Study	Reasons for exclusion
1. Shike M. Diet and lifestyle in the prevention of colorectal cancer: an overview. *Am J Med*. 1999;106(1a):11S-15S; discussion 50S-51S. doi:10.1016/s0002-9343(98)00340-4	Inadequate study design: Not a SR, nor is a MA included.
2. Bergström A, Pisani P, Tenet V, Wolk A, Adami HO. Overweight as an avoidable cause of cancer in Europe. *Int J Cancer*. 2001;91(3):421-430. doi:10.1002/1097-0215(200002)9999:9999<::aid-ijc1053>3.0.co;2-t	Inadequate summary estimate statistics and exposure definition: Overweight - attributable proportion.
3. Terry MB, Neugut AI, Bostick RM, et al. Risk factors for advanced colorectal adenomas: A pooled analysis. *Cancer Epidemiology Biomarkers & Prevention*. 2002;11(7):622-629.	Inadequate outcome: adenomas.
4. Chen K, Qiu JL, Zhang Y, Zhao YW. Meta analysis of risk factors for colorectal cancer. *World J Gastroenterol*. 2003;9(7):1598-1600. doi:10.3748/wjg.v9.i7.1598	Inadequate exposure: Exposure not excess adiposity defined by BMI.
5. Giovannucci E. Diet, body weight, and colorectal cancer: A summary of the epidemiologic evidence. *Journal of Womens Health & Gender-Based Medicine*. 2003;12(2):173-182. doi:10.1089/154099903321576574	Inadequate study design: Not a SR, nor is a MA included.
6. John BJ, Irukulla S, Abulafi AM, Kumar D, Mendall MA. Systematic review: adipose tissue, obesity and gastrointestinal diseases. *Aliment Pharmacol Ther*. 2006;23(11):1511-1523. doi:10.1111/j.1365-2036.2006.02915.x	Inadequate study design: MA not included; Exposure not access adiposity defined by BMI.
7. Bazensky I, Shoobridge-Moran C, Yoder LH. Colorectal cancer: an overview of the epidemiology, risk factors, symptoms, and screening guidelines. *Medsurg Nurs*. 2007;16(1):46-51; quiz 52.	Inadequate study design: Not a SR, nor is a MA included.
8. McCloskey CA, Wilson MA, Hughes SJ, Eid GM. Laparscopic colorectal surgery is safe in the high-risk patient: A NSQIP risk-adjusted analysis. *Surgery*. 2007;142(4):594-597. doi:10.1016/j.surg.2007.07.020	Inadequate study design: Not a SR, nor is a MA included.
9. Miles L. Physical activity and the prevention of cancer: a review of recent findings. *Nutrition Bulletin*. 2007;32(3):250-282. doi:10.1111/j.1467-	Inadequate study design: Not a SR, nor is a MA included.

3010.2007.00653.x	
10. Harriss DJ, Atkinson G, Batterham A, et al. Lifestyle factors and colorectal cancer risk (2): a systematic review and meta-analysis of associations with leisure-time physical activity. *Colorectal Dis*. 2009;11(7):689-701. doi:10.1111/j.1463-1318.2009.01767.x	Inadequate exposure: Primary exposure is physical-activity.
11. Li KS, Yao SP, Liu SY, Wang BY, Mao DW. Genetic polymorphisms of interleukin 8 and risk of ulcerative colitis in the Chinese population. *Clinica Chimica Acta*. 2009;405(1-2):30-34. doi:10.1016/j.cca.2009.03.053	Not a SR, nor is a MA included; Exposure not access adiposity defined by BMI.
12. Renehan AG, Soerjomataram I, Tyson M, et al. Incident cancer burden attributable to excess body mass index in 30 European countries. *Int J Cancer*. 2010;126(3):692-702. doi:10.1002/ijc.24803	Not a SR, nor is a MA included; Inadequate summary estimate statistics – population attributable risk.
13. Lee YJ, Myung SK, Cho B, et al. Adiposity and the risk of colorectal adenomatous polyps: a meta-analysis. *Cancer Causes & Control*. 2011;22(7):1021-1035. doi:10.1007/s10552-011-9777-9	Inadequate outcome: polyps.
14. Ben QW, An W, Jiang Y, et al. Body Mass Index Increases Risk for Colorectal Adenomas Based on Meta-analysis. *Gastroenterology*. 2012;142(4):762-772. doi:10.1053/j.gastro.2011.12.050	Inadequate outcome: adenomas.
15. Hong S, Cai Q, Chen D, Zhu W, Huang W, Li Z. Abdominal obesity and the risk of colorectal adenoma: a meta-analysis of observational studies. *Eur J Cancer Prev*. 2012;21(6):523-531. doi:10.1097/CEJ.0b013e328351c775	Inadequate outcome: adenomas.
16. Okabayashi K, Ashrafian H, Hasegawa H, et al. Body Mass Index Category as a Risk Factor for Colorectal Adenomas: A Systematic Review and Meta-Analysis. *American Journal of Gastroenterology*. 2012;107(8):1175-1185. doi:10.1038/ajg.2012.180	Inadequate outcome: adenomas.
17. Wang D, Zheng W, Wang SM, et al. Estimation of cancer incidence and mortality attributable to overweight, obesity, and physical inactivity in China. *Nutr Cancer*. 2012;64(1):48-56. doi:10.1080/01635581.2012.630166	Not a SR. Inadequate summary estimate statistics: attributable fraction.
18. Win AK, Macinnis RJ, Hopper JL, Jenkins MA. Risk	

prediction models for colorectal cancer: a review. *Cancer Epidemiol Biomarkers Prev*. 2012;21(3):398-410. doi:10.1158/1055-9965.epi-11-0771	Not a SR, nor is a MA included: risk prediction models.
19. Bardou M, Barkun AN, Martel M. Obesity and colorectal cancer. *Gut*. 2013;62(6):933-947. doi:10.1136/gutjnl-2013-304701	Inadequate study design: Not a SR, nor is a MA included.
20. Omata F, Deshpande GA, Ohde S, Mine T, Fukui T. The association between obesity and colorectal adenoma: systematic review and meta-analysis. *Scandinavian Journal of Gastroenterology*. 2013;48(2):136-146. doi:10.3109/00365521.2012.737364	Inadequate outcome: adenomas.
21. Rasool S, Kadla SA, Rasool V, Ganai BA. A comparative overview of general risk factors associated with the incidence of colorectal cancer. *Tumour Biol*. 2013;34(5):2469-2476. doi:10.1007/s13277-013-0876-y	Inadequate exposure: adiposity not explored as a potential risk factor.
22. Coe PO, O'Reilly DA, Renehan AG. Excess adiposity and gastrointestinal cancer. *Br J Surg*. 2014;101(12):1518-1531; discussion 1531. doi:10.1002/bjs.9623	Inadequate study design: Not a SR, nor is a MA included.
23. Haque TR, Bradshaw PT, Crockett SD. Risk Factors for Serrated Polyps of the Colorectum. *Digestive Diseases and Sciences*. 2014;59(12):2874-2889. doi:10.1007/s10620-014-3277-1	Inadequate outcome: polyps.
24. Joshi RK, Kim WJ, Lee SA. Association between obesity-related adipokines and colorectal cancer: a case-control study and meta-analysis. *World J Gastroenterol*. 2014;20(24):7941-7949. doi:10.3748/wjg.v20.i24.7941	Inadequate exposure: obesity-related adipokines.
25. Laiyemo AO. The risk of colonic adenomas and colonic cancer in obesity. *Best Practice & Research Clinical Gastroenterology*. 2014;28(4):655-663. doi:10.1016/j.bpg.2014.07.007	Inadequate study design: Not a SR, nor is a MA included.
26. Ma GK, Ladabaum U. Personalizing colorectal cancer screening: a systematic review of models to predict risk of colorectal neoplasia. *Clin Gastroenterol Hepatol*. 2014;12(10):1624-34.e1. doi:10.1016/j.cgh.2014.01.042	SR of prediction models.
27. Singh S, Singh PP, Murad MH, Singh H, Samadder NJ.	

Prevalence, risk factors, and outcomes of interval colorectal cancers: a systematic review and meta-analysis. *Am J Gastroenterol*. 2014;109(9):1375-1389. doi:10.1038/ajg.2014.171	Inadequate outcomes: interval CRC.
28. Chen Q, Wang J, Yang J, et al. Association between adult weight gain and colorectal cancer: a dose-response meta-analysis of observational studies. *Int J Cancer*. 2015;136(12):2880-2889. doi:10.1002/ijc.29331	Inadequate exposure: weight gain.
29. Dolatkhah R, Somi MH, Kermani IA, et al. Increased colorectal cancer incidence in Iran: a systematic review and meta-analysis. *BMC Public Health*. 2015;15:997. doi:10.1186/s12889-015-2342-9	Inadequate summary estimate statistics: incidence of CRC.
30. Karahalios A, English DR, Simpson JA. Weight change and risk of colorectal cancer: a systematic review and meta-analysis. *Am J Epidemiol*. 2015;181(11):832-845. doi:10.1093/aje/kwu357	Inadequate exposure: weight change.
31. Keum N, Greenwood DC, Lee DH, et al. Adult weight gain and adiposity-related cancers: a dose-response meta-analysis of prospective observational studies. *J Natl Cancer Inst*. 2015;107(2). doi:10.1093/jnci/djv088	Inadequate exposure: weight gain.
32. Keum N, Lee DH, Kim R, Greenwood DC, Giovannucci EL. Visceral adiposity and colorectal adenomas: dose-response meta-analysis of observational studies. *Annals of Oncology*. 2015;26(6):1101-1109. doi:10.1093/annonc/mdu563	Inadequate exposure: visceral adiposity; Inadequate outcome: adenomas.
33. Schlesinger S, Lieb W, Koch M, et al. Body weight gain and risk of colorectal cancer: a systematic review and meta-analysis of observational studies. *Obesity Reviews*. 2015;16(7):607-619. doi:10.1111/obr.12286	Inadequate exposure: weight gain.
34. Thrift AP, Gong J, Peters U, et al. Mendelian Randomization Study of Body Mass Index and Colorectal Cancer Risk. *Cancer Epidemiology Biomarkers & Prevention*. 2015;24(7):1024-1031. doi:10.1158/1055-9965.epi-14-1309	Inadequate study design: Not a SR, nor is a MA included.
35. Benn M, Tybjaerg-Hansen A, Smith GD, Nordestgaard BG. High body mass index and cancer risk-a Mendelian randomisation study. *European*	Inadequate study design: Not a SR, nor is a MA included.

Journal of Epidemiology. 2016;31(9):879-892. doi:10.1007/s10654-016-0147-5	
36. De Ridder J, Julián-Almárcegui C, Mullee A, et al. Comparison of anthropometric measurements of adiposity in relation to cancer risk: a systematic review of prospective studies. *Cancer Causes Control*. 2016;27(3):291-300. doi:10.1007/s10552-015-0709-y	Inadequate study design: combination of different measures of adiposity. No MA was included.
37. Lennon H, Sperrin M, Badrick E, Renehan AG. The Obesity Paradox in Cancer: a Review. *Current Oncology Reports*. 2016;18(9). doi:10.1007/s11912-016-0539-4	Inadequate study design: Not a SR, nor is a MA included.
38. Mysuru Shivanna L, Urooj A. A Review on Dietary and Non-Dietary Risk Factors Associated with Gastrointestinal Cancer. *J Gastrointest Cancer*. 2016;47(3):247-254. doi:10.1007/s12029-016-9845-1	Inadequate study design: Not a SR, nor is a MA included.
39. Pischon T, Nimptsch K. Obesity and Risk of Cancer: An Introductory Overview. *Recent Results Cancer Res*. 2016;208:1-15. doi:10.1007/978-3-319-42542-9_1	Inadequate study design: Not a SR, nor is a MA included.
40. Psaltopoulou T, Ntanasis-Stathopoulos I, Tzanninis IG, Kantzanou M, Georgiadou D, Sergentanis TN. Physical Activity and Gastric Cancer Risk: A Systematic Review and Meta-Analysis. *Clinical Journal of Sport Medicine*. 2016;26(6):445-464. doi:10.1097/jsm.0000000000000316	Inadequate exposure: physical activity.
41. Usher-Smith JA, Walter FM, Emery JD, Win AK, Griffin SJ. Risk Prediction Models for Colorectal Cancer: A Systematic Review. *Cancer Prevention Research*. 2016;9(1):13-26. doi:10.1158/1940-6207.capr-15-0274	Inadequate summary estimate statistics: risk prediction models.
42. Bailie L, Loughrey MB, Coleman HG. Lifestyle Risk Factors for Serrated Colorectal Polyps: A Systematic Review and Meta-analysis. *Gastroenterology*. 2017;152(1):92-104. doi:10.1053/j.gastro.2016.09.003	Inadequate outcome: polyps.
43. Brenner DR, Poirier AE, Grundy A, Khandwala F, McFadden A, Friedenreich CM. Cancer incidence attributable to excess body weight in Alberta in 2012. *CMAJ Open*. 2017;5(2):E330-e336.	

doi:10.9778/cmajo.20160039	Not a SR, nor is a MA included; Inadequate summary estimate statistics: attributable fraction.
44. Fardet A, Druesne-Pecollo N, Touvier M, Latino-Martel P. Do alcoholic beverages, obesity and other nutritional factors modify the risk of familial colorectal cancer? A systematic review. *Crit Rev Oncol Hematol*. 2017;119:94-112. doi:10.1016/j.critrevonc.2017.09.001	Inadequate outcome/summary estimate: familial CRC.
45. Gandomani HS, Yousefi SM, Aghajani M, et al. Colorectal cancer in the world: incidence, mortality and risk factors. *Biomedical Research and Therapy*. 2017;4(10):1656-1675. doi:10.15419/bmrat.v4i10.372	Inadequate study design: Not a SR, nor is a MA included.
46. Grosso G, Bella F, Godos J, et al. Possible role of diet in cancer: systematic review and multiple meta-analyses of dietary patterns, lifestyle factors, and cancer risk. *Nutrition Reviews*. 2017;75(6):405-419. doi:10.1093/nutrit/nux012	Inadequate exposure: diet.
47. Himbert C, Delphan M, Scherer D, Bowers LW, Hursting S, Ulrich CM. Signals from the Adipose Microenvironment and the Obesity-Cancer Link-A Systematic Review. *Cancer Prev Res (Phila)*. 2017;10(9):494-506. doi:10.1158/1940-6207.capr-16-0322	Inadequate exposure.
48. Jayasekara H, Reece JC, Buchanan DD, et al. Risk factors for metachronous colorectal cancer or polyp: A systematic review and meta-analysis. *J Gastroenterol Hepatol*. 2017;32(2):301-326. doi:10.1111/jgh.13476	Inadequate outcome: metachronous CRC or polyp.
49. Jensen BW, Gamborg M, Gogenur I, Renehan AG, Sorensen TIA, Baker JL. Childhood body mass index and height in relation to site-specific risks of colorectal cancers in adult life. *European Journal of Epidemiology*. 2017;32(12):1097-1106. doi:10.1007/s10654-017-0289-0	Inadequate study design: Not a SR, nor is a MA included.
50. Krishna SG, Hussan H, Cruz-Monserrate Z, Conteh LF, Mumtaz K, Conwell DL. A review of the impact of obesity on common gastrointestinal malignancies. *Integr Cancer Sci Ther*. 2017;4(1). doi:10.15761/icst.1000223	Inadequate study design: Not a SR, nor is a MA included.
51. Kyrgiou M, Kalliala I, Markozannes G, et al. Adiposity and cancer at major anatomical sites: umbrella	

review of the literature. *Bmj*. 2017;356:j477. doi:10.1136/bmj.j477	
52. Oines M, Helsingen LM, Bretthauer M, Emilsson L. Epidemiology and risk factors of colorectal polyps. *Best Practice & Research Clinical Gastroenterology*. 2017;31(4):419-424. doi:10.1016/j.bpg.2017.06.004	Inadequate study design: Not a SR, nor is a MA included.
53. Schlesinger S, Aleksandrova K, Abar L, et al. Adult weight gain and colorectal adenomas-a systematic review and meta-analysis. *Ann Oncol*. 2017;28(6):1217-1229. doi:10.1093/annonc/mdx080	Not a SR, nor is a MA included; Inadequate outcome: polyps.
54. Theodoratou E, Timofeeva M, Li X, Meng X, Ioannidis JPA. Nature, Nurture, and Cancer Risks: Genetic and Nutritional Contributions to Cancer. *Annu Rev Nutr*. 2017;37:293-320. doi:10.1146/annurev-nutr-071715-051004	Inadequate exposure: weight gain. Inadequate outcome: adenomas.
55. Xue K, Li FF, Chen YW, Zhou YH, He J. Body mass index and the risk of cancer in women compared with men: a meta-analysis of prospective cohort studies. *Eur J Cancer Prev*. 2017;26(1):94-105. doi:10.1097/cej.0000000000000231	Inadequate study design: Not a SR, nor is a MA included.
56. Zheng J, Zhao M, Li J, et al. Obesity-associated digestive cancers: A review of mechanisms and interventions. *Tumour Biol*. 2017;39(3):1010428317695020. doi:10.1177/1010428317695020	Inadequate summary estimate statistics: Pooled relative risk ratio.
57. Carr PR, Alwers E, Bienert S, et al. Lifestyle factors and risk of sporadic colorectal cancer by microsatellite instability status: a systematic review and meta-analyses. *Ann Oncol*. 2018;29(4):825-834. doi:10.1093/annonc/mdy059	Inadequate study design: Not a SR, nor is a MA included.
58. Choi EK, Park HB, Lee KH, et al. Body mass index and 20 specific cancers: re-analyses of dose-response meta-analyses of observational studies. *Annals of Oncology*. 2018;29(3):749-757. doi:10.1093/annonc/mdx819	Inadequate outcome: sporadic colorectal cancer by microsatellite instability status.
59. Gu MJ, Huang QC, Bao CZ, et al. Attributable causes of colorectal cancer in China. *BMC Cancer*. 2018;18(1):38. doi:10.1186/s12885-017-3968-z	Inadequate study design: Umbrella review.
60. Hidayat K, Yang CM, Shi BM. Body fatness at an	

early age and risk of colorectal cancer. *Int J Cancer*. 2018;142(4):729-740. doi:10.1002/ijc.31100<br><br>61. Islami F, Goding Sauer A, Miller KD, et al. Proportion and number of cancer cases and deaths attributable to potentially modifiable risk factors in the United States. *CA Cancer J Clin*. 2018;68(1):31-54. doi:10.3322/caac.21440<br><br>62. Peng L, Weigl K, Boakye D, Brenner H. Risk Scores for Predicting Advanced Colorectal Neoplasia in the Average-risk Population: A Systematic Review and Meta-analysis. *American Journal of Gastroenterology*. 2018;113(12):1788-1800. doi:10.1038/s41395-018-0209-2<br><br>63. Rezende LFM, Arnold M, Rabacow FM, et al. The increasing burden of cancer attributable to high body mass index in Brazil. *Cancer Epidemiol*. 2018;54:63-70. doi:10.1016/j.canep.2018.03.006<br><br>64. Wienecke A, Neuhauser H, Kraywinkel K, Barnes B. Cancers Potentially Preventable through Excess Weight Reduction in Germany in 2010. *Obes Facts*. 2018;11(5):400-412. doi:10.1159/000490150<br><br>65. Wong MCS, Chan CH, Cheung W, et al. Association between investigator-measured body-mass index and colorectal adenoma: a systematic review and meta-analysis of 168,201 subjects (adenoma and BMI at the time of colonscopy). *European Journal of Epidemiology*. 2018;33(1):15-26. doi:10.1007/s10654-017-0336-x<br><br>66. Anderson AS, Caswell S, Mowat C, Strachan JA, Steele RJC. Lifestyle in patients at increased risk of colorectal cancer. *Journal of Human Nutrition and Dietetics*. 2019;32(5):570-577. doi:10.1111/jhn.12663<br><br>67. Karczewski J, Begier-Krasinska B, Staszewski R, Poplawska E, Gulczynska-Elhadi K, Dobrowolska A. Obesity and the Risk of Gastrointestinal Cancers. *Digestive Diseases and Sciences*. 2019;64(10):2740-2749. doi:10.1007/s10620-019-05603-9<br><br>68. Lopez-Suarez A. Burden of cancer attributable to obesity, type 2 diabetes and associated risk factors. *Metabolism-Clinical and Experimental*. 2019;92:136-	Inadequate study design: Not a SR, nor is a MA included.<br><br>Inadequate study design: Not a SR, nor is a MA included.<br><br>Inadequate study design: Not a SR, nor is a MA included.<br><br>Inadequate outcome: neoplasia.<br><br>Inadequate study design: Not a SR, nor is a MA included.<br><br>Inadequate study design: Not a SR, nor is a MA included.<br><br>Inadequate outcome: adenomas.<br><br>Inadequate study design: Not a SR, nor is a MA included.

146. doi:10.1016/j.metabol.2018.10.013	Inadequate study design: Not a SR, nor is a MA included.
69. McGeoch L, Saunders CL, Griffin SJ, et al. Risk Prediction Models for Colorectal Cancer Incorporating Common Genetic Variants: A Systematic Review. *Cancer Epidemiol Biomarkers Prev*. 2019;28(10):1580-1593. doi:10.1158/1055-9965.epi-19-0059	Inadequate study design: Not a SR, nor is a MA included.
70. Soltani G, Poursheikhani A, Yassi M, Hayatbakhsh A, Kerachian M, Kerachian MA. Obesity, diabetes and the risk of colorectal adenoma and cancer. *Bmc Endocrine Disorders*. 2019;19(1). doi:10.1186/s12902-019-0444-6	Inadequate study design: Not a SR, nor is a MA included – risk prediction models.
71. Wakamatsu M, Sugawara Y, Zhang S, Tanji F, Tomata Y, Tsuji I. Weight change since age 20 and incident risk of obesity-related cancer in Japan: a pooled analysis of the Miyagi Cohort Study and the Ohsaki Cohort Study. *International Journal of Cancer*. 2019;144(5):967-980. doi:10.1002/ijc.31743	Inadequate study design: Not a SR, nor is a MA included.
72. Weihrauch-Blüher S, Schwarz P, Klusmann JH. Childhood obesity: increased risk for cardiometabolic disease and cancer in adulthood. *Metabolism*. 2019;92:147-152. doi:10.1016/j.metabol.2018.12.001	Inadequate exposure: weight change.
73. Xu C, Liu Y, Zhang C, et al. An overview on the methodological and reporting quality of dose-response meta-analysis on cancer prevention. *J Cancer Res Clin Oncol*. 2019;145(5):1201-1211. doi:10.1007/s00432-019-02869-4	Inadequate study design: Not a SR, nor is a MA included.
74. Yang T, Li X, Montazeri Z, et al. Gene-environment interactions and colorectal cancer risk: An umbrella review of systematic reviews and meta-analyses of observational studies. *Int J Cancer*. 2019;145(9):2315-2329. doi:10.1002/ijc.32057	Inadequate study design: Not a SR, nor is a MA included.
75. Breau G, Ellis U. Risk Factors Associated With Young-Onset Colorectal Adenomas and Cancer: A Systematic Review and Meta-Analysis of Observational Research. *Cancer Control*. 2020;27(1):1073274820976670. doi:10.1177/1073274820976670	Inadequate study design: Not a SR, nor is a MA included. Inadequate exposure: gene-environment interaction.
76. Bull CJ, Bell JA, Murphy N, et al. Adiposity, metabolites, and colorectal cancer risk: Mendelian	

randomization study. *Bmc Medicine*. 2020;18(1). doi:10.1186/s12916-020-01855-9	Inadequate outcome: young-onset adenomas/CRC.
77. Gausman V, Dornblaser D, Anand S, et al. Risk Factors Associated With Early-Onset Colorectal Cancer. *Clinical Gastroenterology and Hepatology*. 2020;18(12):2752-+. doi:10.1016/j.cgh.2019.10.009	Inadequate study design: Not a SR, nor is a MA included.
78. Lega IC, Lipscombe LL. Review: Diabetes, Obesity, and Cancer-Pathophysiology and Clinical Implications. *Endocr Rev*. 2020;41(1). doi:10.1210/endrev/bnz014	Inadequate study design: Not a SR, nor is a MA included. Inadequate outcome: young-onset adenomas/CRC.
79. Low EE, Demb J, Liu L, et al. Risk Factors for Early-Onset Colorectal Cancer. *Gastroenterology*. 2020;159(2):492-+. doi:10.1053/j.gastro.2020.01.004	Inadequate study design: Not a SR, nor is a MA included.
80. Ochs-Balcom HM, Kanth P, Farnham JM, Abdelrahman S, Cannon-Albright LA. Colorectal cancer risk based on extended family history and body mass index. *Genetic Epidemiology*. 2020;44(7):778-784. doi:10.1002/gepi.22338	Inadequate study design: Not a SR, nor is a MA included. Inadequate outcome: young-onset adenomas/CRC.
81. Qiang JK, Lipscombe LL, Lega IC. Association between diabetes, obesity, aging, and cancer: review of recent literature. *Translational Cancer Research*. 2020;9(9):5743-5759. doi:10.21037/tcr.2020.03.14	Inadequate study design: Not a SR, nor is a MA included.
82. Solans M, Chan DSM, Mitrou P, Norat T, Romaguera D. A systematic review and meta-analysis of the 2007 WCRF/AICR score in relation to cancer-related health outcomes. *Ann Oncol*. 2020;31(3):352-368. doi:10.1016/j.annonc.2020.01.001	Inadequate study design: Not a SR, nor is a MA included.
83. Weihe P, Spielmann J, Kielstein H, Henning-Klusmann J, Weihrauch-Blueher S. Childhood Obesity and Cancer Risk in Adulthood. *Current Obesity Reports*. 2020;9(3 N1-Klusmann, Jan-Henning/M-9655-2015 Weihrauch-Bluher, Susann/0000-0001-7399-223X; Klusmann, Jan-Henning/0000-0002-1070-0727 3):204-212. doi:10.1007/s13679-020-00387-w	Inadequate exposure: WCRF/AICR score.
84. Zhang X, Gill D, He Y, et al. Non-genetic biomarkers and colorectal cancer risk: Umbrella review and evidence triangulation. *Cancer Med*.	Inadequate study design: Not a SR, nor is a MA included.

2020;9(13):4823-4835. doi:10.1002/cam4.3051	
85. Zhang YB, Pan XF, Chen J, et al. Combined lifestyle factors, incident cancer, and cancer mortality: a systematic review and meta-analysis of prospective cohort studies. *Br J Cancer*. 2020;122(7):1085-1093. doi:10.1038/s41416-020-0741-x	Inadequate study design: Umbrella review.
86. Bandi P, Minihan AK, Siegel RL, et al. Updated Review of Major Cancer Risk Factors and Screening Test Use in the United States in 2018 and 2019, with a Focus on Smoking Cessation. *Cancer Epidemiol Biomarkers Prev*. Published online May 19, 2021. doi:10.1158/1055-9965.epi-20-1754	Inadequate outcome: cancer mortality.
87. Campbell PT, Lin Y, Bien SA, et al. Association of Body Mass Index With Colorectal Cancer Risk by Genome-Wide Variants. *Jnci-Journal of the National Cancer Institute*. 2021;113(1):38-47. doi:10.1093/jnci/djaa058	Inadequate study design: Not a SR, nor is a MA included.
88. Cho S, Shin A. Population Attributable Fraction of Established Modifiable Risk Factors on Colorectal Cancer in Korea. *Cancer Res Treat*. 2021;53(2):480-486. doi:10.4143/crt.2019.742	Inadequate study design: Not a SR, nor is a MA included.
89. Enwerem N, Cho MY, Demb J, et al. Systematic Review of Prevalence, Risk Factors, and Risk for Metachronous Advanced Neoplasia in Patients With Young-Onset Colorectal Adenoma. *Clinical Gastroenterology and Hepatology*. 2021;19(4):680-+. doi:10.1016/j.cgh.2020.04.092	Inadequate study design: Not a SR, nor is a MA included.
90. Han F, Wu G, Zhang S, Zhang J, Zhao Y, Xu J. The association of Metabolic Syndrome and its Components with the Incidence and Survival of Colorectal Cancer: A Systematic Review and Meta-analysis. *Int J Biol Sci*. 2021;17(2):487-497. doi:10.7150/ijbs.52452	Inadequate outcome: Metachronous Advanced Neoplasia in Patients With Young-Onset Colorectal Adenoma.
91. Heo JW, Kim SE, Sung MK. Sex Differences in the Incidence of Obesity-Related Gastrointestinal Cancer. *Int J Mol Sci*. 2021;22(3). doi:10.3390/ijms22031253	Inadequate exposure: metabolic syndrome; obesity not defined by BMI.
92. Liu B, Giffney HE, Arthur RS, Rohan TE, Dannenberg AJ. Cancer Risk in Normal Weight Individuals with Metabolic Obesity: A Narrative Review. *Cancer Prev Res (Phila)*. 2021;14(5):509-520. doi:10.1158/1940-	

6207.capr-20-0633<br><br>93. O'Sullivan DE, Sutherland RL, Town S, et al. Risk Factors for Early-Onset Colorectal Cancer: A Systematic Review and Meta-analysis. *Clin Gastroenterol Hepatol*. Published online January 29, 2021:S1542-3565(21)00087-2. doi:10.1016/j.cgh.2021.01.037<br><br>94. Saadati HM, Okhovat B, Khodamoradi F. Incidence and Risk Factors of Colorectal Cancer in the Iranian Population: a Systematic Review *J Gastrointest Cancer*. 2021;52(2):414-421. doi:10.1007/s12029-020-00574-x<br><br>95. Sawicki T, Ruszkowska M, Danielewicz A, Niedźwiedzka E, Arłukowicz T, Przybyłowicz KE. A Review of Colorectal Cancer in Terms of Epidemiology, Risk Factors, Development, Symptoms and Diagnosis. *Cancers (Basel)*. 2021;13(9). doi:10.3390/cancers13092025<br><br>96. Scheruebl H. Excess Body Weight and Gastrointestinal Cancer Risk. *Visceral Medicine*. Published online 2021. doi:10.1159/000515444<br><br>97. Suzuki S, Goto A, Nakatochi M, et al. Body mass index and colorectal cancer risk: A Mendelian randomization study. *Cancer Science*. 2021;112(4):1579-1588. doi:10.1111/cas.14824<br><br>98. Shen X, Wang Y, Zhao R, et al. Metabolic syndrome and the risk of colorectal cancer: a systematic review and a meta-analysis. *Int. J. Colorectal Dis.* 2021 1999; 36(10):2215-2225; doi: 10.1007/s00384-021-03974-y.	Inadequate study design: Not a SR, nor is a MA included.<br><br>Inadequate study design: Not a SR, nor is a MA included.<br><br>Inadequate outcome: early-onset CRC.<br><br>Inadequate study design: No MA included. Inadequate summary estimate statistics: adiposity not quantitatively assessed as a risk factor.<br><br>Inadequate study design: Not a SR, nor is a MA included.<br><br>Inadequate study design: Not a SR, nor is a MA included.<br><br>Inadequate study design: Not a SR, nor is a MA included.<br><br>Inadaquate exposure: BMI and waist measures combined.

Abbreviations: BMI = body-mass index; CC = Colon cancer, CRC = colorectal cancer, MA = meta-analysis, SR = systematic review.
