# Supplementary material for: Is the association of overweight and obesity with colorectal cancer underestimated? An umbrella review of systematic reviews and meta-analyses
Source: Eur J Epidemiol. 2023 Jan 21;38(2):135–44. doi: 10.1007/s10654-022-00954-6 (PMC9905196; doi:10.1007/s10654-022-00954-6)
Supplement: Supplementary file 7 — Supplementary file7 (DOCX 34 KB) [file 10654_2022_954_MOESM7_ESM.docx]

## Supplementary Table 1. Comparison of initial years excluded in the main and sensitivity analysis of cohort studies in the most recent review by Zhang et al. 2021^3^.

| Study | First years of  follow-up  excluded in  main analysis | First years of  follow-up  excluded in  sensitivity analysis | Follow-up  duration  (years) |
| --- | --- | --- | --- |
| Moore et al. 2004^29^ | 4 | 4 | Not reported |
| Engeland et al. 2005^30^ | 0 | 5 | m: 23 |
| Oh et al. 2005^31^ | 0 | 2 | m: ≤9 |
| Rapp et al. 2005^32^ | 1 | 3 | m: 9.9 |
| Larsson et al. 2006^33^ | 0 | 2 | m: 7.1 |
| Lukanova et al. 2006^34^ | 0 | 1 | m: 8.2 |
| Reeves et al. 2007^35^ | 0 | 2 | m: 5.4 |
| Song et al. 2008^36^ | 0 | 5 | m: 8.75 |
| Bassett et al. 2010^37^ | 0 | 2 | m: 14 |
| Laake et al. 2010^38^ | 1 | 1 | m: 23.2 |
| Doubeni et al. 2012^39, a^ | n/a | n/a | n/a (m: <9) |
| Matsuo et al. 2012^40, b^ | n/a | n/a | n/a (m: 11) |
| Renehan et al. 2012^41^ | 0 | 0 | m: <9 |
| Li et al. 2013^42^ | 0 | 1 | m: 11.0 and m: 5.5^d^ |
| Bhaskaran et al. 2014^43^ | 1 | 3 | m: 7.5 |
| Steins Bisschop et al. 2014^44^ | 0 | 2 | M: 6.8 |
| Han et al. 2014^45, c^ | n/a | n/a | n/a |
| Lu et al. 2015^46^ | 0 | 2 | m: 11.3 |
| Kantor et al. 2016^47, c^ | n/a | n/a | n/a (m: 35) |
| Hanyuda et al. 2017^48^ | 0 | 0 | m: >26 |
| Levi et al. 2017^49, c(p20^ | n/a | n/a | n/a (M: 23) |
| Andreasson et al. 2019^50^ | 0 | 0 | M: 21.5 |
| Bjorge et al. 2019^51^ | 0 | 1 | m: 17.6 |
| Liu et al. 2019^52^ | 0 | 4 | M: 13.9 |
| Noh et al. 2020^53^ | 1 | 1 | m: 17 |
| Wang et al. 2020^54^ | 1 | 3 | m: 8.95 |

**Abbreviations:** n/a = not Applicable, m = mean, M = median.

^a^ Study not directly assessing the BMI-CRC association.

^b^ Study reported “excluding early cases”, without further explanation.

^c^ Study looking at BMI exclusively at early adulthood.

^d^ Study includes two different cohorts.
