# Supplementary material for: Is the association of overweight and obesity with colorectal cancer underestimated? An umbrella review of systematic reviews and meta-analyses
Source: Eur J Epidemiol. 2023 Jan 21;38(2):135–44. doi: 10.1007/s10654-022-00954-6 (PMC9905196; doi:10.1007/s10654-022-00954-6)
Supplement: Supplementary file 8 — Supplementary file8 (DOCX 29 KB) [file 10654_2022_954_MOESM8_ESM.docx]

| **Supplementary Table 2**. Comparison of results from main and sensitivity analysis of cohort studies in the most recent review by Zhang et al. 2021^3^ | | | | | | | |
| --- | --- | --- | --- | --- | --- | --- | --- |
| Study | **Exposure** | **Type** | **Sex** | **Main Analysis** | | **Sensitivity Analysis** | |
|  |  |  |  | **Years**  **Excluded** | **HR (95% CI)** | **Years**  **Excluded** | **HR (95% CI)** |
| Lukanova et al. 2006^34^ | ≥30 vs 18.5-24.9 kg/m^2^ | CRC | M | 0 | 1.61 (0.95 to 2.65) | 1 | 1.77 (1.04 to 2.95) |
| Larsson et al. 2006^33^ | ≥30 vs <23 kg/m^2^ | CRC | M | 0 | 1.54 (1.08 to 2.21) | 2 | 1.65 (1.09 to 2.51) |
| Song et al. 2008^36^ | ≥30 vs 21-22.9 kg/m^2^ | CC | F | 0 | 2.18 (1.43 to 3.33) | 5 | 2.43 (1.40 to 4.23) |
| Song et al. 2008^36^ | ≥30 vs 21-22.9 kg/m^2^ | RC | F | 0 | 0.91 (0.55 to 1.52) | 5 | 1.13 (0.57 to 2.24) |
| Liu et al. 2019^52^ | ≥30 vs 18.5-22.9 kg/m^2^ | CRC | F | 0 | 1.93 (1.15 to 3.25) | 4 | 1.77 (1.00 to 3.11) |
| Liu et al. 2019^52^ | 25-29.9 vs 18.5-22.9 kg/m^2^ | CRC | F | 0 | 1.20 (1.05 to 1.38) | 4 | 1.42 (0.83 to 2.43) |
| Song et al. 2008^36^ | 1 kg/m^2^ increase | CC | F | 0 | 1.05 (1.02 to 1.08) | 5 | 1.05 (1.02 to 1.09) |
| Song et al. 2008^36^ | 1 kg/m^2^ increase | RC | F | 0 | 1.00 (0.97 to 1.03) | 5 | 1.03 (0.99 to 1.06) |
| Bhaskaran et al. 2014^43^ | 5 kg/m^2^ increase | CC | MF | 1 | 1.10 (1.07 to 1.13) | 3 | 1.12 (1.06 to 1.18) |
| Bhaskaran et al. 2014^43^ | 5 kg/m^2^ increase | RC | MF | 1 | 1.04 (1.00 to 1.08) | 3 | 1.05 (0.97 to 1.15) |
| Liu et al. 2019^52^ | 5 kg/m^2^ increase | CRC | F | 0 | 1.20 (1.05 to 1.38) | 4 | 1.20 (1.02 to 1.40) |
| Wang et al. 2020^54^ | 5 kg/m^2^ increase | CRC | MF | 1 | 1.18 (1.06 to 1.31) | 3 | 1.21 (1.13 to 1.30) |
| Reeves et al. 2007^35^ | 10 kg/m^2^ increase | CRC | F | 0 | 1.00 (0.92 to 1.08) | 2 | 0.98 (0.89 to 1.09) |

**Abbreviations**: BMI = body mass index, CC = colon cancer, CI = confidence interval, CRC = colorectal cancer, F = female, HR = hazard ratio, M = male, RC = rectal cancer, WHO = World Health Organization.
